# Polymorphisms of *ITGA9* Gene and Their Correlation with Milk Quality Traits in Yak (*Bos grunniens*)

**DOI:** 10.3390/foods13111613

**Published:** 2024-05-22

**Authors:** Mengfan Zhang, Xita Zha, Xiaoming Ma, Yongfu La, Xian Guo, Min Chu, Pengjia Bao, Ping Yan, Xiaoyun Wu, Chunnian Liang

**Affiliations:** 1 Key Laboratory of Yak Breeding Engineering of Gansu Province, Lanzhou Institute of Husbandry and Pharmaceutical Sciences, Chinese Academy of Agricultural Sciences, Lanzhou 730050, China; zmf13664139695@163.com (M.Z.); maxiaoming@caas.cn (X.M.); layongfu@caas.cn (Y.L.); guoxian@caas.cn (X.G.); chumin@caas.cn (M.C.); baopengjia@caas.cn (P.B.); pingyanlz@163.com (P.Y.); wuxiaoyun@caas.cn (X.W.); 2 Key Laboratory of Animal Genetics and Breeding on Tibetan Plateau, Ministry of Agriculture and Rural Affairs, Lanzhou 730050, China; 3 Qinghai Province Qilian County Animal Husbandry and Veterinary Workstation, Qilian 810400, China; zhaxita@163.com

**Keywords:** single-nucleotide polymorphism, association analysis, haplotypes, yak milk

## Abstract

A single-nucleotide polymorphism (SNP) is a genome-level trait that arises from a variation in a single nucleotide, leading to diversity in DNA sequences. SNP screening is commonly used to provide candidate genes for yak breeding efforts. Integrin Subunit Alpha 9 (*ITGA9*) is an integrin protein. It plays an important role in cell adhesion, signalling, and other processes. The aim of this study was to discuss the association between genetic polymorphisms in the *ITGA9* gene and milk quality traits and to identify potential molecular marker loci for yak breeding quality. We genotyped 162 yaks using an Illumina Yak cGPS 7K liquid chip and identified the presence of polymorphisms at nine SNP loci in the *ITGA9* gene of yaks. The results showed that the mutant genotypes in the loci g.285,808T>A, g.306,600T>C, and g.315,413C>T were positively correlated with the contents of casein, protein, total solids (TS), and solid nonfat (SNF) in yak milk. In other loci, heterozygous genotypes had a positive correlation with nutrient content in yak milk. Then, two *ITGA9* haplotype blocks were constructed based on linkage disequilibrium, which facilitated a more accurate screening of *ITGA9* as a candidate gene for yak milk quality improvement. In conclusion, we identified SNPs and haplotype blocks related to yak milk quality traits and provided genetic resources for marker-assisted selection in yak breeding.

## 1. Introduction

Yaks (*Bos grunniens*) live on the Qinghai–Tibet Plateau and its neighbouring alpine and subalpine areas, mainly relying on natural pasture to draw the nutrients needed for growth, and are an important livestock species indispensable to the sustainable development of the local animal husbandry economy [1]. Yak milk is a real non-polluting green food, has great value for development and utilisation when raw [2], can be made into a variety of dairy products, and is one of the main sources of protein for local people. Compared with cow milk, yak milk has significant advantages in terms of milk protein, milk fat, and amino acid content [3]. The proteins in yak milk are divided into two main groups: casein and whey proteins. Compared with other milk sources, yak milk has a higher total whey protein content [4], provides essential amino acids for growth and development, and is rich in a variety of proteins involved in immunity [5]. Peptides released from casein in yak milk have a variety of biological activities. They can exhibit antioxidant, antihypertensive, antibacterial, antithrombotic, and immunomodulatory activities. It is a typical dietary protein with some functional properties [6,7,8,9]. The composition of yak milk varies with seasonal grass growth and climate change [3]. The nutrient content of yak milk is highest during lactation, with 5.5–7.5% fat, 4.0–5.9% protein, and 4.0–5.9% lactose content, but the fat content continues to increase in the later stages of lactation [3]. Therefore, yak milk is known as natural milk concentrate.

Heat stress effects may be deleterious to milk yield and quality [10], and have an effect on the fat content and protein content of milk [11]. *ITGA9* is a heat shock protein that is associated with body temperature during heat stress [12] and plays a crucial role in environmental stress adaptation and thermal homeostasis [13]. Vittoria Bisutti et al. [14] found the presence of *ITGA9* in Protothera-infected milk using the somatic cell (SC) transcriptome in their study of subclinical mastitis in dairy cows. *ITGA9* is associated with mastitis in cows due to Protothera infection and affects milk quality. The *ITAG9* gene is located on chromosome 22, which encodes the alpha integrin. This integrin, which is less well studied, promotes accelerated cell migration and regulates a variety of biological functions such as angiogenesis, lymphangiogenesis, cancer cell proliferation, and migration [15]. Fibroblasts secrete VEGFC ligands through the *ITGA9* receptor to promote angiogenic pathways and ensure the transport of nutrients for lactation [16]. Also, the *ITGA9* gene has been studied in cancer therapy. Luydmila A Mostovich et al. [15] found that the *ITGA9* gene is present in normal human breast tissue. In breast cancer, the expression level of *ITGA9* changes. Thus, we analysed the SNPs associated with casein, protein, SNF, and other traits related to milk quality on the *ITGA9* gene.

Currently, SNP loci are commonly used to characterise growth traits in livestock but are less frequently used in milk samples. Studies on milk samples have mainly been used to explore correlations in milk yield and less frequently in studying milk quality. There is almost a gap in the research on yak milk.

In this study, yak milk was used as a research object. We relied on the typing results obtained from resequencing and obtained SNP loci related to milk quality by Genome-Wide Association Studies (GWAS). After screening, we found multiple SNPs on the *ITAG9* gene. By comparing the phenotypic differences among different genotypes of SNP loci, we can reveal the genetic mechanisms associated with these traits and provide new ideas and methods for yak breeding and improvement.

## 2. Materials and Methods

### 2.1. Ethics Approval

All the animal experiments were approved by the Lanzhou Institute of Animal Husbandry and Pharmaceutical Sciences of the Chinese Academy of Agricultural Sciences (CAAS) with grant number 1610322020018.

### 2.2. Sample Collection of Test Animals

The samples were collected from Gannan yaks in the natural summer pasture in Xiahe County, Gannan Tibetan Autonomous Prefecture, Gansu Province. A total of 162 female yaks with similar milk yield and parity ranging from 2 to 3 were selected, and the animals were naturally grazed without artificial supplemental feeding.

#### 2.2.1. Milk Samples

The female yaks were hand-milked in the morning of lactation. The udder was first scrubbed, then fully massaged to give appropriate stimulation; next, milking was performed by the two-handed grasping method. The milk was milked and mixed, and a portion of the milk sample was collected in a 50 mL centrifuge tube to obtain a separate milk sample from each yak. The samples were stored at 4 °C for milk quality determination.

#### 2.2.2. Tissue Samples

Selecting the ear margin area with fewer blood vessels, thinner skin, and greater ease of sampling, a small portion of the ear margin tissue samples from 162 Gannan yaks were collected using U-shaped ear forceps, which were operated quickly to avoid stress and discomfort to the yaks. The samples were placed in freezing tubes and stored in liquid nitrogen. The samples were brought back to the laboratory and stored in a −80 °C refrigerator for DNA extraction.

### 2.3. Milk Composition Analysis

The collected yak milk was measured using the MilkoScanTM FT120 Milk Composition Analyzer (FUCHS Analytical Instruments Ltd., Hellerup, Denmark). The measurements included fat, protein, lactose, casein, solid nonfat (SNF), acidity, and total solids (TS) content.

### 2.4. DNA Extraction

The magnetic bead method was used to extract DNA from the tissue samples, using the Magnetic Animal Tissue Genomic DNA Kit (DP341, Tiangen Biochemical Technology Co., Ltd., Beijing, China). The magnetic bead method of DNA extraction was mainly based on the properties of magnetic beads and the affinity between surface modification molecules and DNA, which realised the efficient enrichment and purification of DNA. The DNA samples obtained from the extraction were first used for concentration determination by Qubit fluorescence quantification. We used 1% agarose gel electrophoresis to detect the integrity of the DNA samples. By observing the electrophoresis profile, we could visually determine whether there were quality problems, such as degradation and breakage of the DNA samples, to ensure the integrity of the samples. Only after the above quality control could confirmed qualified DNA samples be used in the subsequent library preparation process.

### 2.5. Genotyping

A total of 162 Gannan yaks were genotyped using Illumina Yak cGPS 7K (Illumina, Huazhi Biotechnology Co., Ltd., Changsha, China) liquid microarrays. The genomic locations of the SNPs were derived from the assembly of the yak reference genome Bosgru v3.0 (GCA_005887515.1). cGPS genotyping technology mainly relies on precise sequencing of liquid-capture targets, and the technique is capable of accurately capturing the genomic sequence of a target region, followed by sequencing and typing analysis. cGPS genotyping technology involves specific probe designs for target intervals, genome-wide DNA fragmentation, the addition of sequencing junctions, hybridisation of probes to the target intervals, binding of the target intervals of magnetic beads to liquid-capture probes, amplification and enrichment of the captured target intervals, second-generation sequencing of the captured target intervals, and genotypic variant analysis of the captured target intervals. Through these steps, detailed information about the genetic makeup of an individual can be obtained to further understand the relationship between genes and phenotypes.

### 2.6. Statistical Analysis

Homozygosity (HO), heterozygosity (HE), number of effective alleles (NE), polymorphism (PIC), genotypes, and minor allele frequency (MAF) were calculated for the nine loci using the GDICALL (http://www.msrcall.com/gdicall.aspx (accessed on 12 March 2024)) online software, and *p*-values for the Hardy–Weinberg test were calculated.

We used one-way analysis of variance (ANOVA) in IBM SPSS Statistics 23 (IBM, Armonk, NY, USA) to explore the relationship between *ITGA9* gene polymorphisms and yak milk quality traits.

### 2.7. LD Linkage Disequilibrium and Haplotype Analyses

Linkage disequilibrium and inferred haplotypes of *ITGA9* SNPs in yaks were estimated by Haploview software (v 4.2) [17]. Haplotypes are combinations of alleles at multiple loci that are coinherited on the same chromosome. The minor allele frequency (MAF) was <0.001 for SNPs and failed to achieve Hardy–Weinberg equilibrium (HWE; *p* < 0.05), which excluded them from the haplotype structure.

### 2.8. KEGG Signalling Pathway Enrichment Analysis

The *ITGA9* gene was analysed using KOBAS 3.0 to screen which signalling pathways the *ITGA9* gene was present in.

## 3. Results

### 3.1. Genotype Results for ITGA9 and Genetic Parameter Analysis of the Loci in Yak

**Figure 1:**
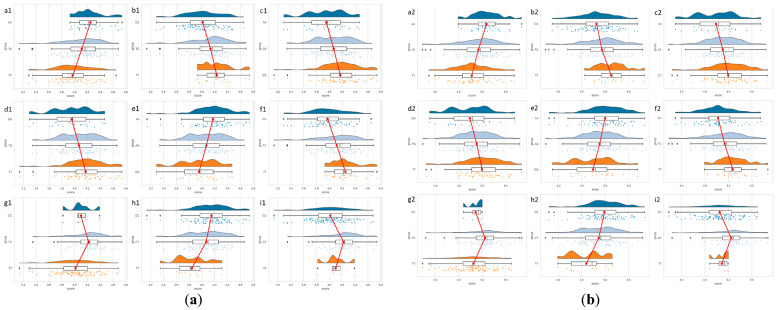
Raincloud plots. (**a**) Distribution of genotypes of nine SNPs (casein). (**b**) Distribution of genotypes of nine SNPs (protein). a: g.285,808T>A; b: g.285,847T>C; c: g.285,867C>A; d: g.285,916T>G; e: g.285,952A>G; f: g.285,965T>C; g: g.306,600T>C; h: g.306,635C>T; i: g.315,413C>T. The red lines represent the mean values of milk quality traits corresponding to the genotypes. The three different coloured dots represent the distribution of the data.

Figure 1 shows the distribution of genotypes related to the nine SNPs in the *ITGA9* gene for major traits in yaks. As can be seen from the figure, the distribution of SNPs is relatively uniform.

Table 1 provides a description of the SNPs and related population parameters in the *ITGA9* gene. The full length of the yak *ITGA9* gene is about 412.99 kbp, and many variants exist. In this study, a total of nine SNP sites were screened for the presence of polymorphisms. All loci had calculated PIC values between 0.25 and 0.50, were moderately polymorphic in the population analysed, and had MAF above 5%. The genotype distribution of all loci was in Hardy–Weinberg equilibrium.

Among *ITGA9* g.285,808T>A, g.285,867C>A, g.285,916T>G, g.285,952A>G, and g.285,965T>C, the genotype frequencies of TA, CA, TG, AG, and TC were the highest, 0.494, 0.500, 0.500, 0.500, and 0.494, respectively, indicating that heterozygosity was dominant in these five SNP loci. Among the genotype frequencies of g.285,847T>C, g.306,600T>C, g.306,635C>T, and g.315,413C>T, the highest frequencies of CC, TT, CC, and CC types were 0.463, 0.629, 0.475 and 0.623, respectively, which indicated that the homozygous type was predominant among these four SNP loci.

In g.285,847T>C and g.285,965T>C, the genotype frequency of CC was 0.679 and 0.593, respectively, indicating that the mutant allele was predominant in this locus. Unmutated alleles predominated in seven loci (g.285,808T>A, g.285,867C>A, g.285,916T>G, g.285,952A>G, g.306,600T>C, g.306,635C>T and g.315,413C>T).

### 3.2. Association Analysis of ITGA9 Genotypes with Yak Milk Quality Traits

Table 2 shows the association of individual loci with yak milk quality traits analysed by genotyping the data of SNPs. Nine SNP loci in the *ITAG9* gene (g.285,808T>A, g.285,847T>C, g.285,867C>A, g.285,916T>G, g.285,952A>G, g.285,965T>C, g.306,600T>C, g.306,635C>T, and g.315,413C>T) are all associated with proteins and caseins in milk.

The g.285,808T>A locus was associated with casein, protein, total solids (TS), and solid nonfat (SNF) content (*p* < 0.05). The AA homozygous genotypes were higher than the TA and TT genotypes in terms of casein and protein content (*p* < 0.05), higher than the TT genotypes in TS and SNF content (*p* < 0.05), and higher than the TT genotypes in terms of casein and protein (*p* < 0.01). The TA heterozygous genotypes had an increase in casein content compared to the TT genotype (*p* < 0.01). The mutant genotypes of this locus had higher nutrient content than the wild genotypes (*p* < 0.05). The g.285,847T>C locus was associated with casein, protein, solid nonfat (SNF), and acidity traits (*p* < 0.05). The casein and protein contents of TT homozygous genotypes were higher than that of CC genotypes (*p* < 0.01) but no difference was found between the TT and TC genotypes. The TT genotypes had higher SNF and acidity than the TC and CC genotypes (*p* < 0.05).

The g.285,867C>A, g.285,916T>G, and g.285,952A>G loci showed similar correlations with milk quality. Correlations were found with casein and protein content in yak milk (*p* < 0.05). Among the three loci, the casein and protein contents of wild homozygous genotypes (CC, TT, AA) were higher than those of mutant homozygous genotypes (AA, GG, GG) (*p* < 0.01). The casein content of wild homozygous genotypes was higher than heterozygous genotypes (*p* < 0.05). However, there was no difference in milk protein content between wild homozygous genotypes and heterozygous genotypes (*p* > 0.05).

Three loci, g.285,965T>C, g.306,600T>C, and g.315,413C>T, were associated with casein, protein, TS, and SNF contents in yak milk (*p* < 0.05). The casein and protein contents of TT homozygous genotypes and TC mutant heterozygous genotypes in g.285,965T>C were higher than CC genotypes (*p* < 0.01). TS was higher in TC heterozygotes and TT homozygotes than CC genotypes (*p* < 0.05). However, there were no differences in protein, SNF, and TS contents between the TT and TC genotypes (*p* > 0.05). In g.306,600T>C, the TT mutant homozygous and TC mutant heterozygous genotypes had higher casein, protein, and SNF contents than the TT genotypes with extremely significant differences (*p* < 0.01). The TS content of the TC type was higher than that of the TT genotypes (*p* < 0.05), and no difference was found with the CC genotypes (*p* > 0.05). The contents of all components were higher in both homozygous and heterozygous mutant genotypes than in the wild type (*p* < 0.05). This indicated that the mutant types had higher-quality yak milk. The g.315,413C>T locus was associated with casein, protein, TS, and SNF traits (*p* < 0.05). Casein, protein, and SNF were higher in the CT mutant genotype than in the CC genotype (*p* < 0.01). TS content was higher in the CT than in the CC genotype (*p* < 0.05), and no difference was found with the TT genotype (*p* > 0.05). The nutritional contents in mutant heterozygous genotypes were higher than in the wild type (*p* < 0.05).

The g.306,635C>T locus was correlated (*p* < 0.05) with casein, protein, and SNF traits. Casein and protein were higher in the CC homozygous and CT mutant heterozygous genotypes than in the TT genotypes (*p* < 0.01). SNF content was higher in the CC homozygous and CT heterozygous genotypes than in the TT genotypes (*p* < 0.05). There was no difference between CC homozygous genotypes and CT mutant heterozygous genotypes (*p* > 0.05).

Among these nine loci, the mutant genotypes at g.285,808T>A, g.306,600T>C, and g.315,413C>T loci had higher milk nutrient content than the wild type (*p* < 0.05). At other loci, mutant heterozygous genotypes were higher than mutant homozygous genotypes (*p* < 0.05), but there was no difference between them and wild homozygous genotypes (*p* > 0.05). Overall, the mutations brought positive effects on yak milk quality, and the milk quality of mutant yaks was higher.

### 3.3. LD Analysis and Haplotype Analysis

**Figure 2:**
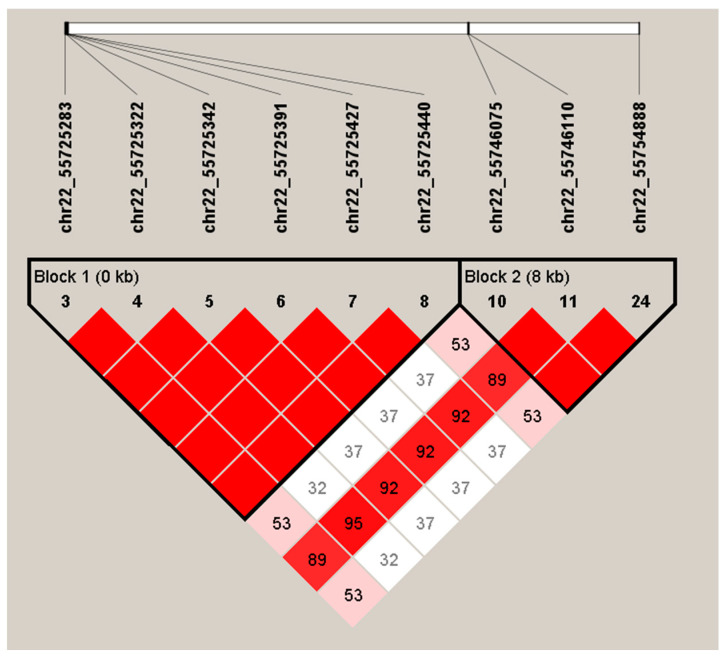
Linkage disequilibrium structure of the *ITGA9* gene in yaks.

**Figure 3:**
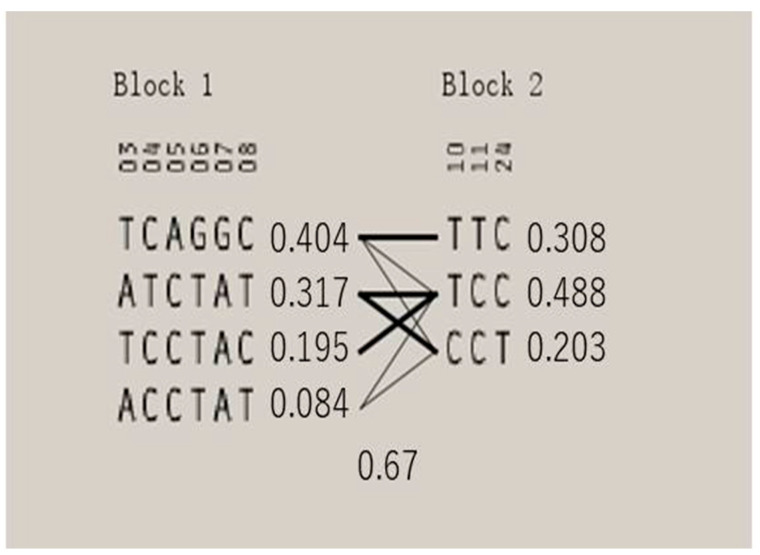
Haplotype blocks defined by the nine SNPs genotyped in the yak *ITGA9* gene.

The results of LD analysis using Haploview software are shown in Figure 2 and Figure 3. The chain imbalance analysis of *ITGA9* resulted in a set of nine SNPs covering about 29.6 kb, with an average distance between SNPs of 3.7 kb. Two LD regions were identified in *ITGA9*, and Haplogroup blocks 1 and 2 were adjacent to each other. Block 1 is the largest and consists of six SNPs. It includes four haplotype combinations: H1 (TCAGGC), H2 (ATCTAT), H3 (TCCTAC), and H4 (ACCTAT), with frequencies of 0.404, 0.317, 0.195, and 0.084, respectively. The second haplotype block includes three SNPs with three different combinations of haplotypes, H5 (TTC), H6 (TCC), and H7 (CCT). The frequencies were 0.308, 0.488, and 0.203, respectively.

### 3.4. KEGG Signalling Pathway Annotation Analysis

Enrichment analysis of the *ITGA9* gene using KOBAS 3.0 revealed that the *ITGA9* gene was enriched in the PI3K-Akt signaling pathway, ECM–receptor interaction, and other signaling pathways affecting milk quality.

## 4. Discussion

Yak milk is a rare dairy product from the Tibetan Plateau region, which supplies the nutritional needs of the local people. Studies have shown that in the absence of fruits and vegetables in the Tibetan Plateau region, the nomadic herdsmen of the region have never shown symptoms of vitamin or mineral deficiencies [18]. The uniqueness of yak milk is that it contains a high concentration of nutrients, including protein, essential amino acids, fat, lactose, and minerals [19]. In particular, the content of protein, fat, and minerals is much higher than that of regular cow’s milk [20]. It can satisfy the needs of newborn babies, the elderly, or specific groups of people [20]. In addition, the immunoglobulin and lactoferrin in yak milk can enhance human immunity and promote gastrointestinal health [21]. Its unique Conjugated Linoleic Acid (CLA) has a stimulating effect on improving bone density, promoting bone development, strengthening muscle tissues, helping reduce fat accumulation, and preventing cancer and cardiovascular disease [22].

Protein is the core component of milk, and its content and quality are now one of the key indicators of milk quality. Especially at the peak of lactation, the proteins secreted by the mammary glands account for up to 43% of the total protein synthesised in the body, with milk proteins occupying a dominant position, accounting for 90% of the total [23,24]. This kind of milk protein is a kind of high-quality protein with comprehensive nutrition, which is rich in many kinds of essential amino acids needed by the human body and easily digested and absorbed by the human body. It is worth mentioning that the rich proteins in yak milk not only help to enhance the body’s immunity but also have the biological function of promoting the absorption of minerals such as calcium (Ca) and phosphorus (P) [25].

Milk protein mainly consists of casein and whey protein; casein is the main protein in cow’s milk, which is synthesised by mammary tissues themselves as a phosphorus-containing acidic protein [5,26], accounting for about 80% of the total protein in cow’s milk [27]. Among the types of casein, β-casein has the highest content. The active casein peptide in yak milk has a significant inhibitory effect on angiotensin-converting enzyme (ACE), and this inhibitory effect can effectively reduce the generation of angiotensin [28,29], regulating vasodilation and lowering blood pressure; thus, yak milk is an important source of anti-hypertensive peptides. Casein from yak milk is a typical dietary protein with high nutritional value and can be used as a versatile ingredient in a variety of value-added functional meals [6].

The solid nonfat (abbreviated as SNF) is the milk solids remaining in the milk after it has been defatted. It consists mainly of milk proteins, lactose, and inorganic components that provide the body with key nutrients other than fat. These components play an essential role in supporting the body’s normal growth, development, and metabolism. At the same time, because of the removal of the fat portion, solid nonfat is particularly suitable for those who need to control fat intake, such as obese patients and patients with high blood fat. Therefore, solid nonfat can also be used as an important food ingredient in a wide range of food manufacturing applications. The proteins in milk have beneficial nutritional properties that are key to transforming milk into cheese and other dairy products. Therefore, a high concentration of protein improves the quality and overall value of milk.

Single-nucleotide polymorphisms (SNPs) are the simplest form of DNA variation between individuals [30]. They may be responsible for diversity among individuals, genome evolution, most common family traits, inter-individual differences in drug response, etc. SNP loci can be used as selective markers for product quality improvement and are now widely used in animal breeding [31]. Rosemarie Weikard et al. [32] screened polymorphisms in the promoter region of the *PPARGC1* gene and obtained 11 polymorphic loci, whereas there was an association between one of the SNP sites and milk fat production, suggesting that this gene may participate in milk fat synthesis. Binyam S Dagnachew et al. [33] demonstrated that exon 12 deletion in *CSN1S1* found in Norwegian dairy goats was associated with milk quantity and quality (*p* < 0.05). *GHR* polymorphisms were associated with the fat, urea, and lactose content of sheep’s milk. Variations in *IGF1* were associated with the content of milk proteins and casein [34]. A total of 21 SNPs associated with milk production traits were identified in the *PKLR* gene [35]. A total of 13 SNPs were identified in the *HTR1B* gene and demonstrated to have a genetic effect on milk fatty acids in dairy cows [36]. *ITGA9* is located in the PI3K-Akt signalling pathway, which contains several genes involved in milk quality regulation. *EEF1D* can promote the synthesis of milk fat and milk quality through the regulation of PI3K-Akt signalling [37]. *GPAM* gene polymorphisms affect milk quality by influencing triglyceride metabolism in the mammary epithelial cells of dairy cows [38]. The functional 3′ UTR polymorphism of *FADS2* affects milk composition by modifying Mir-744 binding [39]. The *FADS2* gene may have an effect on milk fatty acid composition by influencing liver metabolism [40]. The *SLC2A2* gene affects milk quality by influencing the transfer of nutrients from the blood to the emulsion [41]. There is an increasing number of studies using SNP loci to explore genetic effects affecting milk quality, but further development is needed in yaks.

In the present experiment, nine SNP loci of *ITGA9* in yaks were found to affect mainly milk protein, casein, and SNF in yaks, which were higher in the mutant populations in g.285,808T>A, g.285,847T>C, g.306,600T>C, and g.315,413C>T than in the wild type (*p* < 0.05). In the rest of the loci, the contents of the wild homozygous populations were higher than those of the mutant homozygous populations, but no differences were found with the mutant heterozygous populations. Overall, the mutation of SNPs improved yak milk quality.

In this experiment, we found that there were nine SNP loci in yak *ITGA9* that positively influenced yak milk quality, and the wild and heterozygous genotypes of the six loci, g.285,867C>A, g.285,916T>G, g.285,847T>C, g.285,952A>G, g.285,965T>C, and g.306,635C>T, were positively associated with the nutrient content of milk (*p* < 0.05). The g.285,808T>A locus and mutant genotypes of the g.315,413C>T locus had positive effects on the casein, protein, TS, and SNF content of yak milk (*p* < 0.05). The g.306,600T>C locus had a positive effect on the casein, protein, TS and SNF content of yak milk (*p* < 0.05). Overall, mutations at all nine SNP loci positively affected yak milk traits to varying degrees.

In general, interlocking SNP loci are of interest because of the large number of LDs between their SNPs [42]. The large number of LDs suggests a close association between these alleles. In this study, two haplotype blocks were identified on the *ITGA9* gene, which may be a key factor affecting milk quality. Haplotype combinations involving multiple linked SNP loci may provide more precise information for association analysis than single SNP markers [43,44,45]. By comparing the haplotypes of different individuals or breeds, the genetic relationships between them can be investigated, revealing genetic variation that affects trait expression. It helps to better understand the genetic basis of animal traits.

The results of this study confirmed the effect of the *ITGA9* gene on milk quality traits in yak populations. There are fewer studies on the *ITGA9* gene in milk quality, but according to the KEGG results, the *ITGA9* gene was enriched in signalling pathways involved in lactogenesis, such as PI3K-Akt [37,46], and in combination with the correlation with milk quality phenotypes and strong genetic effects obtained in the present study, we can consider the *ITGA9* gene as a candidate for the study of milk quality. The novel SNPs in the *ITGA9* gene could be used as potential genetic markers for genetic improvement in yak breeding programs. This may provide new insights for improving yak milk quality traits through selection strategies.

## 5. Conclusions

In this study, nine SNP loci with polymorphisms present in the *ITGA9* gene were explored and analysed, and we found that all nine SNP loci were moderately polymorphic and conformed to Hardy–Weinberg equilibrium. Thus, stable inheritance is possible. Correlation showed that all of these loci were associated with the casein and protein content of yak milk (*p* < 0.05). Some loci were also correlated with SNF and TS (*p* < 0.05). These loci had a positive effect on yak milk quality. In this study, we also screened the existence of two haplotype groups of the *ITGA9* gene, which further enhanced the accuracy of *ITGA9* as a candidate marker for yak milk quality enhancement. We will further investigate the effects of these SNPs on other economic traits in yaks. In conclusion, our study provides important genetic variations that can be used as potential markers for optimising milk production traits in yaks. It lays the foundation for the selective breeding of yaks.

## Figures and Tables

**Table 1:** Description of the markers mapping to the *ITGA9* genes and associated population parameters in yaks.

SNPs	Position	Genotypic Frequencies			Allelic Frequencies		He	Ne	PIC	HW*pval*	MAF	Alleles
g.285,808	Intron	AA	TA	TT	A	T	0.483	1.934	0.366	0.772	0.407	T:(A)
		0.160	0.494	0.346	0.407	0.593						
g.285,847	Intron	CC	TC	CC	C	T	0.436	1.773	0.341	0.911	0.321	C:(T)
		0.463	0.432	0.105	0.679	0.321						
g.285,867	Intron	AA	CA	CC	A	C	0.477	1.911	0.363	0.533	0.392	C:(A)
		0.142	0.500	0.358	0.392	0.608						
g.285,916	Intron	GG	TG	TT	G	T	0.477	1.911	0.363	0.533	0.392	T:(G)
		0.142	0.500	0.358	0.392	0.608						
g.285,952	Intron	AA	AG	GG	A	G	0.477	1.911	0.363	0.533	0.392	A:(G)
		0.358	0.500	0.142	0.608	0.392						
g.285,965	Intron	CC	TC	TT	C	T	0.483	1.934	0.366	0.772	0.407	C:(T)
		0.346	0.494	0.160	0.593	0.407						
g.306,600	Intron	CC	TC	TT	C	T	0.324	1.480	0.272	0.404	0.204	T:(C)
		0.031	0.346	0.629	0.204	0.796						
g.306,635	Intron	CC	CT	TT	C	T	0.422	1.730	0.333	0.498	0.302	C:(T)
		0.475	0.444	0.080	0.698	0.302						
g.315,413	Intron	CC	CT	TT	C	T	0.324	1.480	0.272	0.404	0.204	C:(T)
		0.623	0.346	0.031	0.796	0.204						

**Table 2:** Correlation analysis between loci in *ITGA9* and milk traits in yak.

	SNP g.285,808T>A						
**Genotype**	**Casein/%**	**Protein/%**	**Fat/%**	**SNF/%**	**Lactose/%**	**Acidity/°** **T**	**TS/%**
AA	4.25 ± 0.22 ^Aa^	5.09 ± 0.31 ^Aa^	6.09 ± 3.2	11.47 ± 0.52 ^a^	4.99 ± 0.16	12.79 ± 1.12	17.44 ± 3.05 ^a^
TA	4.12 ± 0.29 ^Ab^	4.92 ± 0.41 ^ABb^	5.87 ± 2.65	11.26 ± 0.48 ^ab^	4.97 ± 0.17	12.4 ± 1.4	17.03 ± 2.58 ^a^
TT	3.97 ± 0.28 ^Bc^	4.78 ± 0.37 ^Bb^	4.88 ± 2.25	11.18 ± 0.42 ^b^	5 ± 0.14	12.18 ± 1.17	15.91 ± 2.18 ^b^
*p*-Value	*p* = 0.000	*p* = 0.003	*p* = 0.052	*p* = 0.037	*p* = 0.619	*p* = 0.137	*p* = 0.013
	SNPs g.285,847T>C						
**Genotype**	**Casein/%**	**Protein/%**	**Fat/%**	**SNF/%**	**Lactose/%**	**Acidity/°** **T**	**TS/%**
CC	4.01 ± 0.28 ^Bb^	4.82 ± 0.37 ^Bb^	5.15 ± 2.3	11.22 ± 0.41 ^b^	5 ± 0.15	12.24 ± 1.19 ^b^	16.23 ± 2.26
TC	4.13 ± 0.3 ^ABa^	4.93 ± 0.41 ^ABab^	6.07 ± 2.97	11.25 ± 0.53 ^b^	4.96 ± 0.17	12.38 ± 1.41 ^b^	17.22 ± 2.87
TT	4.23 ± 0.24 ^Aa^	5.13 ± 0.32 ^Aa^	5.33 ± 2.49	11.53 ± 0.44 ^a^	4.98 ± 0.13	13.1 ± 0.99 ^a^	16.72 ± 2.46
*p*-Value	*p* = 0.003	*p* = 0.009	*p* = 0.104	*p* = 0.043	*p* = 0.342	*p* = 0.044	*p* = 0.068
	SNPs g.285,867C>A						
**Genotype**	**Casein/%**	**Protein/%**	**Fat/%**	**SNF/%**	**Lactose/%**	**Acidity/°** **T**	**TS/%**
AA	3.95 ± 0.31 ^Bb^	4.74 ± 0.45 ^B^	4.97 ± 2.58	11.09 ± 0.54	5.01 ± 0.16	12.15 ± 1.3	15.96 ± 2.39
CA	4.07 ± 0.27 ^ABb^	4.88 ± 0.36 ^AB^	5.43 ± 2.38	11.26 ± 0.4	4.99 ± 0.17	12.37 ± 1.25	16.56 ± 2.35
CC	4.17 ± 0.3 ^Aa^	4.99 ± 0.4 ^A^	5.99 ± 2.98	11.34 ± 0.54	4.96 ± 0.13	12.51 ± 1.35	17.21 ± 2.9
*p*-Value	*p* = 0.006	*p* = 0.023	*p* = 0.240	*p* = 0.096	*p* = 0.356	*p* = 0.527	*p* = 0.113
	SNPs g.285,916T>G						
**Genotype**	**Casein/%**	**Protein/%**	**Fat/%**	**SNF/%**	**Lactose/%**	**Acidity/°** **T**	**TS/%**
GG	3.95 ± 0.31 ^Bb^	4.74 ± 0.45 ^B^	4.97 ± 2.58	11.09 ± 0.54	5.01 ± 0.16	12.15 ± 1.3	15.96 ± 2.39
TG	4.07 ± 0.27 ^ABb^	4.88 ± 0.36 ^AB^	5.43 ± 2.38	11.26 ± 0.4	4.99 ± 0.17	12.37 ± 1.25	16.56 ± 2.35
TT	4.17 ± 0.3 ^Aa^	4.99 ± 0.4 ^A^	5.99 ± 2.98	11.34 ± 0.54	4.96 ± 0.13	12.51 ± 1.35	17.21 ± 2.9
*p*-Value	*p* = 0.006	*p* = 0.023	*p* = 0.240	*p* = 0.096	*p* = 0.356	*p* = 0.527	*p* = 0.113
	SNPs g.285,952A>G						
**Genotype**	**Casein/%**	**Protein/%**	**Fat/%**	**SNF/%**	**Lactose/%**	**Acidity/°** **T**	**TS/%**
AA	4.17 ± 0.3 ^Aa^	4.99 ± 0.4 ^A^	5.99 ± 2.98	11.34 ± 0.54	4.96 ± 0.13	12.51 ± 1.35	17.21 ± 2.9
AG	4.07 ± 0.27 ^ABb^	4.88 ± 0.36 ^AB^	5.43 ± 2.38	11.26 ± 0.4	4.99 ± 0.17	12.37 ± 1.25	16.56 ± 2.35
GG	3.95 ± 0.31 ^Bb^	4.74 ± 0.45 ^B^	4.97 ± 2.58	11.09 ± 0.54	5.01 ± 0.16	12.15 ± 1.3	15.96 ± 2.39
*p*-Value	*p* = 0.006	*p* = 0.023	*p* = 0.240	*p* = 0.096	*p* = 0.356	*p* = 0.527	*p* = 0.113
	SNPs g.285,965T>C						
**Genotype**	**Casein/%**	**Protein/%**	**Fat/%**	**SNF/%**	**Lactose/%**	**Acidity/°** **T**	**TS/%**
CC	3.97 ± 0.28 ^Bc^	4.78 ± 0.37 ^Bc^	4.88 ± 2.25	11.18 ± 0.42 ^b^	5 ± 0.14	12.18 ± 1.17	15.91 ± 2.18 ^b^
TC	4.12 ± 0.29 ^Ab^	4.92 ± 0.41 ^ABb^	5.87 ± 2.65	11.26 ± 0.48 ^ab^	4.97 ± 0.17	12.4 ± 1.4	17.03 ± 2.58 ^a^
TT	4.25 ± 0.22 ^Aa^	5.09 ± 0.31 ^Aa^	6.09 ± 3.2	11.47 ± 0.52 ^a^	4.99 ± 0.16	12.79 ± 1.12	17.44 ± 3.05 ^a^
*p*-Value	*p* = 0.000	*p* = 0.003	*p* = 0.052	*p* = 0.037	*p* = 0.619	*p* = 0.137	*p* = 0.013
	SNPs g.306,600T>C						
**Genotype**	**Casein/%**	**Protein/%**	**Fat/%**	**SNF/%**	**Lactose/%**	**Acidity/°** **T**	**TS/%**
CC	4.1 ± 0.21 ^AB^	4.86 ± 0.16 ^AB^	6.13 ± 3.57	11.14 ± 0.32 ^AB^	5.05 ± 0.15	12.3 ± 0.27	17.28 ± 3.57 ^ab^
TC	4.22 ± 0.26 ^A^	5.06 ± 0.38 ^A^	6.1 ± 2.88	11.4 ± 0.47 ^A^	4.96 ± 0.17	12.64 ± 1.27	17.4 ± 2.77 ^a^
TT	4.01 ± 0.29 ^B^	4.81 ± 0.39 ^B^	5.24 ± 2.44	11.19 ± 0.47 ^B^	4.99 ± 0.15	12.26 ± 1.32	16.3 ± 2.36 ^b^
*p*-Value	*p* = 0.000	*p* = 0.001	*p* = 0.130	*p* = 0.025	*p* = 0.363	*p* = 0.202	*p* = 0.033
	SNPs g.306,635C>T						
**Genotype**	**Casein/%**	**Protein/%**	**Fat/%**	**SNF/%**	**Lactose/%**	**Acidity/°** **T**	**TS/%**
CC	4.15 ± 0.28 ^Aa^	4.99 ± 0.37 ^Aa^	5.77 ± 2.84	11.33 ± 0.47 ^a^	4.96 ± 0.14	12.44 ± 1.32	16.96 ± 2.79
CT	4.07 ± 0.28 ^Aa^	4.86 ± 0.4 ^Aa^	5.44 ± 2.53	11.25 ± 0.47 ^a^	5 ± 0.17	12.43 ± 1.3	16.57 ± 2.44
TT	3.84 ± 0.27 ^Bb^	4.6 ± 0.37 ^Bb^	5.06 ± 2.1	10.97 ± 0.47 ^b^	5.01 ± 0.13	11.9 ± 1.04	15.95 ± 1.98
*p*-Value	*p* = 0.001	*p* = 0.002	*p* = 0.581	*p* = 0.036	*p* = 0.143	*p* = 0.370	*p* = 0.356
	SNPs g.315,413C>T						
**Genotype**	**Casein/%**	**Protein/%**	**Fat/%**	**SNF/%**	**Lactose/%**	**Acidity/°** **T**	**TS/%**
CC	4.01 ± 0.29 ^B^	4.81 ± 0.39 ^B^	5.24 ± 2.44	11.19 ± 0.47 ^B^	4.99 ± 0.15	12.26 ± 1.32	16.3 ± 2.36 ^b^
CT	4.22 ± 0.26 ^A^	5.06 ± 0.38 ^A^	6.1 ± 2.88	11.4 ± 0.47 ^A^	4.96 ± 0.17	12.64 ± 1.27	17.4 ± 2.77 ^a^
TT	4.1 ± 0.21 ^AB^	4.86 ± 0.16 ^AB^	6.13 ± 3.57	11.14 ± 0.32 ^AB^	5.05 ± 0.15	12.3 ± 0.27	17.28 ± 3.57 ^ab^
*p*-Value	*p* = 0.000	*p* = 0.001	*p* = 0.130	*p* = 0.025	*p* = 0.363	*p* = 0.202	*p* = 0.033

Note: Capital letters indicate highly significant differences (*p* < 0.05) and lower-case letters indicate significant differences (*p* < 0.01).

## Data Availability

The data presented in this study are available on request from the corresponding author.
